# The IMPACT framework and implementation for accessible in silico clinical phenotyping in the digital era

**DOI:** 10.1038/s41746-023-00878-9

**Published:** 2023-07-21

**Authors:** Andrew Wen, Huan He, Sunyang Fu, Sijia Liu, Kurt Miller, Liwei Wang, Kirk E. Roberts, Steven D. Bedrick, William R. Hersh, Hongfang Liu

**Affiliations:** 1 Department of AI & Informatics, Mayo Clinic, Rochester, MN 55905 USA; 2 School of Biomedical Informatics, University of Texas Health Science Center, Houston, TX 77030 USA; 3 Department of Medical Informatics and Clinical Epidemiology, Oregon Health & Science University, Portland, OR 97239 USA

**Keywords:** Translational research, Data mining, High-throughput screening

## Abstract

Clinical phenotyping is often a foundational requirement for obtaining datasets necessary for the development of digital health applications. Traditionally done via manual abstraction, this task is often a bottleneck in development due to time and cost requirements, therefore raising significant interest in accomplishing this task via in-silico means. Nevertheless, current in-silico phenotyping development tends to be focused on a single phenotyping task resulting in a dearth of reusable tools supporting cross-task generalizable in-silico phenotyping. In addition, in-silico phenotyping remains largely inaccessible for a substantial portion of potentially interested users. Here, we highlight the barriers to the usage of in-silico phenotyping and potential solutions in the form of a framework of several desiderata as observed during our implementation of such tasks. In addition, we introduce an example implementation of said framework as a software application, with a focus on ease of adoption, cross-task reusability, and facilitating the clinical phenotyping algorithm development process.

## Introduction

**Fig. 1:**
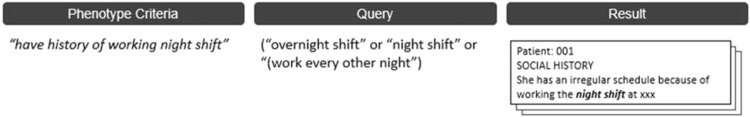
An example NLP-based clinical phenotyping task. An example clinical phenotyping task for determining whether a patient has a history of working night shifts. On the left, we show how such a criterion might be depicted in plain-text. In the center, we show what such a query might look like for text-based applications. On the right, we show a relevant text fragment from a clinical narrative.

The rapid proliferation of the Electronic Health Record (EHR) and the associated availability of voluminous digitized clinical data has led to tremendous interest in the development of digital health applications. Crucial to this is the ability to subset patients using clinical inclusion and exclusion criteria: commonly referred to as clinical phenotyping, patient screening, or cohort retrieval^1,2^ (see Fig. 1). Traditionally conducted manually, there has been great interest in accelerating phenotyping via in-silico means^3,4^. Cross-task generalizable solutions for in-silico phenotyping, however, are not widespread^5^.

In this work, we introduce Intelligent Machine for Patient Accrual and Classification Tasks (IMPACT), a framework and an example implementation highlighting desiderata for accessible and re-usable in-silico phenotyping tools as observed through our efforts in delivering in-silico phenotyping solutions.

### The IMPACT framework for accessible in-silico clinical phenotyping

Variations in task-specific factors such as complexity, required information, and desired results^6^ have hindered implementation of task-generalizable phenotyping solutions^7,8^. Here, we present several desiderata for in-silico phenotyping tools, as well as existing approaches, where applicable.

### Desideratum I: Be infrastructure-flexible and scalable

Adapting software products is generally easier than switching computing infrastructure, necessitating flexibility in data inputs/outputs and computing infrastructure. This can be accomplished through built-in support for various popular setups, for both data repository type (e.g., SQL, Elasticsearch^9^, MongoDB^10^, BigQuery^11^, Fast Health Interoperability Resources (FHIR)^12^ datastores) and model (e.g., Observational Medical Outcomes Partnership (OMOP)^13^ and PCORnet^14^ Common Data Models (CDMs)).

In addition, tools must be scalable as it would otherwise be unfeasible to run phenotyping across largescale datasets without significant engineering effort/time, particularly when involving data sources requiring natural language processing (NLP) or image processing to extract clinical information.

### Desideratum II: Support both ranked score and boolean retrieval schemes

Determining patient classification as a boolean true/false may not always be ideal. Instead, score-based ranking on closeness of match may be appropriate^15^, particularly during algorithm refinement due to missing evidence (e.g., relevant information not present in data sources used). Boolean retrieval, where patients are classified as either fully matching or not matching a given phenotype, fails to produce results when missing evidence is present. Conversely, ranked retrieval will surface patients that may be missing only a subset of the criteria for further review. Boolean retrieval, however, may still be appropriate once an algorithm matures (e.g., for large-scale cohort accrual), necessitating support for both retrieval modes.

Clinical CDMs such as OMOP^13^ and PCORnet^14^ possess boolean retrieval capabilities. Ranked-based retrieval, however, is relatively less prevalent, and approaches focus on unstructured text. Examples of such efforts include the Electronic Medical Record Search Engine (EMERSE)^16^ and Cohort Retrieval Enhanced by the Analysis of TExt (CREATE)^17^ systems, as well as the adoption of various open-source frameworks such as Apache Lucene^18^, Solr^19^, and Elasticsearch^9^ for institution-specific implementations.

### Desideratum III: Support multi-modal retrieval and result integration

Fully determining whether a patient matches a phenotype may not always be possible with the information contained within any single data source, requiring additional data sources, e.g., for information documented in clinical narratives^20–22^ as opposed to within structured EHR data records, or information from radiology images and associated reports^23–25^.

In addition, traditional EHR-based data sources are potentially biased in that underserved/underrepresented populations will be similarly underrepresented in the data, a significant concern for data-driven downstream applications^26–28^. Inclusion of additional data sources helps ameliorate this issue. For instance, if the site doing the in-silico phenotyping is a tertiary medical institution, a substantial amount of history will not be available structurally (e.g., only available via scanned images or clinical text). If only a structured data source is used for phenotyping, the results will be biased as rural/underrepresented populations may have a substantial history captured in text or image^29^ and thus inaccessible to the phenotyping algorithm.

Multi-modal computation of complex phenotype definitions consequently complicates in-silico implementation. Manual overhead is introduced via identification of additional necessary data sources, query refinement to local data representations, scoring, and result integration.

These processes should therefore be supported within the tool itself, rather than being left to manual efforts. While solutions do exist for multi-server querying in the general domain (e.g., cross-server joins in SQL), such solutions tend to be difficult to setup, be limited to a single data type, and have scoring be done on a per-data source basis, thus leading to retrieval not being truly multi-modal.

### Desideratum IV: Support extensions such that textual phenotype definitions can be autonomously converted into local code sets for review

Many phenotype definitions are distributed as textual descriptions^30^. For in-silico phenotyping, these textual descriptors are typically manually translated into equivalent institutional data source-computable representations^31,32^. Similarly, even for those phenotypes distributed as computable representations^33–35^, said representations will typically also need further refinement prior to local use, particularly if natural language processing (NLP) is involved^36^. Such conversions/refinements (e.g., disease names to International Classification of Diseases 10 codes, or appropriate textual variants for NLP-derived data) are typically done over multiple iterations^3^, bottlenecking new algorithm implementation.

Collectively predefining valuesets that correspond to a specific phenotype criterion before distribution of the phenotype definition has been proposed^37^. Usage, however, may not always be feasible for implementing institutions. For instance, while the Logical Observation Identifiers Names and Codes (LOINC) vocabulary is used to codify lab tests, some institutions may use an institution-local code-set without a LOINC mapping. Incorporating standard vocabularies in CDMs such as the OMOP CDM^13^ partially addresses this issue, but requiring usage of the CDM violates Desideratum I, and implementations are non-uniform^5^. In addition, the information required for a phenotyping task may not always be fully representable in the CDM. Explicitly defining such valuesets, while helpful as an initial reference point, will therefore often still require additional manual conversion.

To reduce manual burden, increase mapping reusability, and accelerate the implementation of new phenotype definitions, tools should therefore provide the capability to autonomously convert textual descriptions into local representations. An interface should be provided for abstractors to review/refine conversions. In addition, the capability for individual institutions to implement mappings to local datasets from textual descriptions should be provided. Existing examples of such autonomous mapping systems include Eligibility criteria Information Extraction (EliIE)^30^ and Criteria2Query^38^. General clinical NLP systems such as MedTagger^39^ and the Clinical Text Analysis Knowledge Extraction System (cTAKES)^40^ are also repurposable for this task.

### Desideratum V: Maximize reusability and data reproducibility, minimize technical overhead, and enhance downstream generalizability

The domain expertize of typical users of phenotyping tools differs from those that would possess the knowledge to integrate tools with local data sources, and extract information from said data sources. Ideally, as the latter setup process tends to be the bottlenecking step for in-silico phenotyping algorithm implementation, toolsets should be reusable across multiple phenotyping tasks.

Beyond toolset reusability, however, individual phenotyping projects should also be reusable, from both monoinstitutional and multiinstitutional perspectives. As cohort retrieval is typically only an intermediate, but bottlenecking, step for other downstream applications, the ability to easily reuse identified cohorts is highly desirable to reduce duplicate development/phenotyping efforts^31,41–43^.

In addition, given that data reproducibility has been found critically lacking for datasets^44–47^, there is substantial benefit in centralized storage of both in-silico phenotyping algorithms and retrieved cohorts within a common toolset for later re-use and/or re-execution.

Finally, while cross-institution sharing of retrieved cohorts is unlikely due to privacy concerns, a common framework with sharable definitions will dramatically facilitate multi-institution phenotyping execution, facilitating development and evaluation of cross-institutionally generalizable digital health applications^8,32,48^.

These considerations are one of the motivations behind clinical CDMs such as OMOP^13^ and PCORnet^14^.

### Desideratum VI: Reflect that in-silico phenotyping is an iterative, human-in-the-loop process

The human interpretation and translation process from textual definitions to local data source representations can be highly subjective, leading to inter-abstractor variation both within and without a clinical institution^32,49,50^.

Consequently, iterative definition refinement is required. This may involve manual review by multiple clinical abstractors to identify missing data elements and adjudicate disagreements in definition interpretations, repeating until adequate performance is achieved^51^.

To support such algorithm development, refinement, and implementation processes, tools must therefore support: (a) editing/refining phenotype definitions, (b) surfacing evidence supporting classification for review, and (c) identifying abstraction differences for adjudication.

Graphical user frontends supporting querying against the various clinical common data models (e.g., OHDSI Atlas^52^) support accessible editing phenotyping definitions and reviewing returned results. Such systems, however, typically lack support for presenting supporting evidence and relevance judgement, leading to the development of systems such as PRAI^53^ and CREATE^17^.

### An example IMPACT implementation

Here, we present a full-stack in-silico phenotyping solution implementing these desiderata consisting of:

- A web-based frontend user interface (UI) for phenotyping criteria definition and execution, as well as result relevance judgement and adjudication
- A middleware component supporting cohort management, phenotype definition and abstractor judgement retention, patient evidence retrieval, textual descriptions translation, and job scheduling.
- A backend that performs data source information retrieval and scoring, FHIR mapping, and writes match status, patient scores, and associated evidence to a database.

**Fig. 2:**
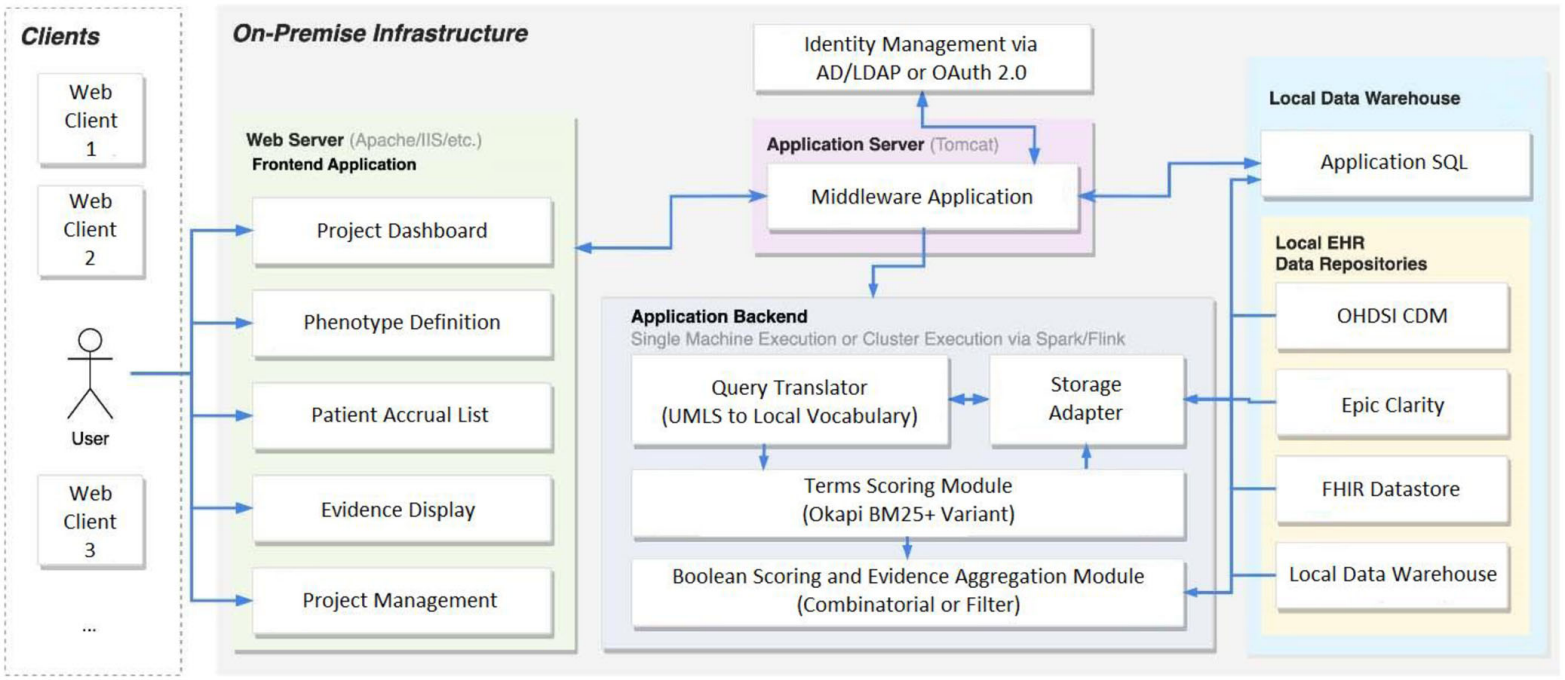
*IMPACT System Architecture*. A Diagram Showing an On-Premise Deployment of IMPACT. Desideratum I is implemented via the Local Data Warehouse, Desideratum II is implemented via the Terms Scoring Module, Desideratum III is implemented via the evidence aggregation module, Desideratum IV is implemented via the query translator, Desideratum V is implemented via the middleware application, and Desideratum VI is implemented via the web frontend.

An overview of the system architecture using an example fully on-premises deployment is provided in Fig. 2. Additional example diagrams using other infrastructure setups can be found on our GitHub https://www.github.com/OHNLP/IMPACT. In the ensuing subsections, we will detail how IMPACT implements our listed desiderata.

### Infrastructurally-agnostic, scalable, ranking-based patient-phenotype matching

To address scalability while maintaining flexibility across differing infrastructure setups, we implemented the backend using Apache Beam^54^, which is usable both across a wide variety of horizontally scaling frameworks, as well as on a single machine. For more details on horizontal scaling and the specific frameworks supported by the example IMPACT implementation, please refer to the Supplementary Information.

For ranked scoring, we leverage a modification of BM25 + ^55,56^ to score patients relative to how well they match the phenotype, where each patient is treated as a “document” and clinical entities such as a diagnosis or a lab test are “tokens” within said “document”. Firstly, leaf criterion (i.e., is not a combinatorial boolean condition such as “must have all of”, “at least *n* of”, “none of”, or similar, but rather a description of a condition, medication, etc.) are grouped such that they are of the same clinical entity type, and BM25+ scoring is run separately for each. Specifically, the base BM25+ score for a given patient *P* and leaf criterion *c*_*i*_ can be calculated as shown in eq. (1):

1 $$BM{25}^{+}({c}_{i},P)={\rm{In}}\left(\frac{N-n({c}_{i})+0.5}{n({c}_{i})+0.5}+1\right)\ast \left(\frac{f({c}_{i},P)\ast ({k}_{1}+1)}{f({c}_{i},P)+{k}_{1}\ast \left(1-b+b\ast \frac{|P|}{avgplen}\right)}+\delta \right)$$

where *N* is the number of patients in the data source, *n*(*c*_*i*_) is the number of patients that leaf criterion *c*_*i*_ matches, *f*(*c*_*i*_, *P*) is the number of distinct records for which patient matches criterion *c*_*i*_, |*P*| is the patient term length (i.e., number of entities of the same clinical data type (condition, medication, etc) as *c*_*i*_), *avgplen* is the average |*P*| across all patients in the cohort. The BM25+ scores of leaf criteria are then combined based on the boolean logic as defined by the phenotype definition. For OR (“must have at least *n* of”), the mean of the top scores of child criteria is used. For AND (“must have all of”), the mean score of all children is used. For NOT (“must not have”), the maximum of all child scores is multiplied by −1. For more details on the BM25+ algorithm, its selection as our default scoring algorithm, and associated hyperparameters, please refer to the Supplementary Information. A Java application programming interface (API) is also provided for implementing custom scoring algorithms.

### Data source flexibility via FHIR conversions, CDM support, and JSON-based plug and play configuration

For IMPACT, we chose to use HL7 Fast Health Interoperability Resources (FHIR) R4^12^ data structures as our internal representation for clinical data. For more details on FHIR and why it was chosen, please refer to the Supplementary Information.

So long as a mapping function can be written to produce FHIR resources, any data source can be used in IMPACT. To facilitate adoption, we supply built-in functions for common use cases. For SQL/JDBC compatible data sources, a configurable mapping function is provided that allows users to specify SQL queries and associated FHIR mappings via JavaScript Object Notation (JSON) config. For on-demand clinical NLP (i.e., artifacts extracted at runtime), we build upon our previous work^57^ to provide a clinical information extraction mapping function that extracts clinical entities to text and converts them^58,59^ to appropriate FHIR resources. Built-in support and mapping functions for the OMOP^13^ (including NLP tables) and PCORnet^14^ CDMs are also provided that allow for immediate, out-of-the-box, use with minimal additional configuration. Custom mapping functions can also be included via implementation of a Java API.

IMPACT supports cross-server data integration by allowing for an arbitrary number of data sources to be queried on any given phenotyping task so long as common patient IDs are used (or can be mapped) and a FHIR mapping function is defined. The data sources and mappings used for scoring are specified as part of a JSON configuration and can be customized on a per-project basis via the frontend GUI. Individual patient scores are computed per-data source and are then combined using a weighted summation (please refer to the Supplementary Information section on BM25+ scoring for more details).

### Autonomous NLP-based conversion of textual phenotype definitions

To generate data source-computable representations from textual definitions, the middleware component contains an integrated MedTagger^39,57^ pipeline to perform named entity recognition and entity linking to Unified Medical Language System (UMLS)^60^ concept codes (CUIs). For more information on the UMLS, coding systems, and the necessity of codeset mapping, please refer to the Supplementary Information. Each leaf criterion (i.e., some clinical entity that is part of the phenotype definition, as opposed to non-leaf criterion, which refers to the boolean logics such as “must have all/one/none of …” that links multiple leaf criterion together) automatically goes through this pipeline to generate a UMLS CUI code set if no computable representations are provided. This process can also be manually triggered by the end user. The UMLS CUIs are then converted to local data source formats depending on data source configurations. IMPACT offers built in mapping to any UMLS source vocabulary, to the OHDSI Athena Vocabulary^61^, as well any UMLS subset for the on-demand NLP data source. In addition, manual mappings from UMLS CUIs can be provided via configuration. End users may also extend our Java API to implement their own mapping function.

The generated representations are then grouped by data source and displayed in the frontend web interface for refinement by clinical abstractors.

### Re-usable infrastructure and phenotype representations and associated implications on data reproducibility and downstream algorithm generalizability

Thus far, we have primarily discussed backend components that must be setup on initial deployment. Once this setup is complete, the system can be re-used across a large variety of phenotyping tasks without additional setup/technical expertize required (unless the addition of more data sources is desired), thus greatly accelerating implementation of new phenotyping algorithms. In addition, common re-usable infrastructure greatly accelerates porting to multiinstitutional settings, facilitating generalizable algorithm development.

The retention of abstractor curated representations of a phenotype by the middleware component enables later re-use. To maximize re-use, users may choose to publicize these collections of representations within the IMPACT platform and share with other users at the same institution.

Central storage of the refined algorithms and datasets on the middleware server also greatly enhances data provenance/reproducibility. Should the algorithm need to be re-ran (e.g., for updated data temporally), the original local representations and associated refinements are retained, as well as a specific record of which datasets/data sources were queried in the original retrieval. Similarly, should it be desired to re-use the retrieved patient cohort itself, the retrieved cohort along with human judgements and associated query metadata is retained for immediate download.

### Human in the loop evidence review and adjudication

**Fig. 3:**
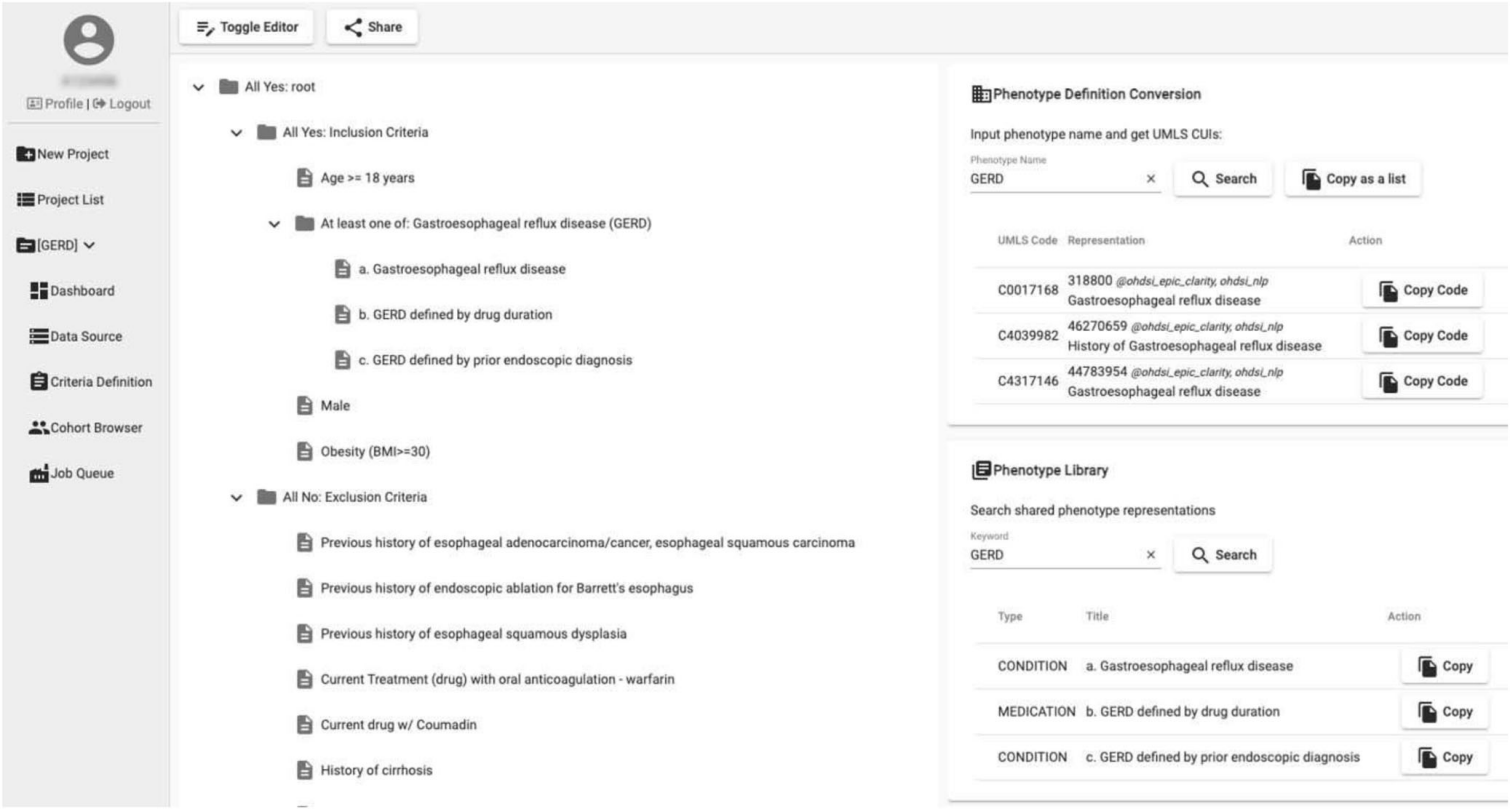
IMPACT phenotype definition page. On the left panel, the user-defined phenotype definition is shown. On the top right, textual definitions can be mapped to datasource-local representations. On the bottom-right, datasource representations for specific criteria that were previously manually curated and shared can be retrieved and reused.

**Fig. 4:**
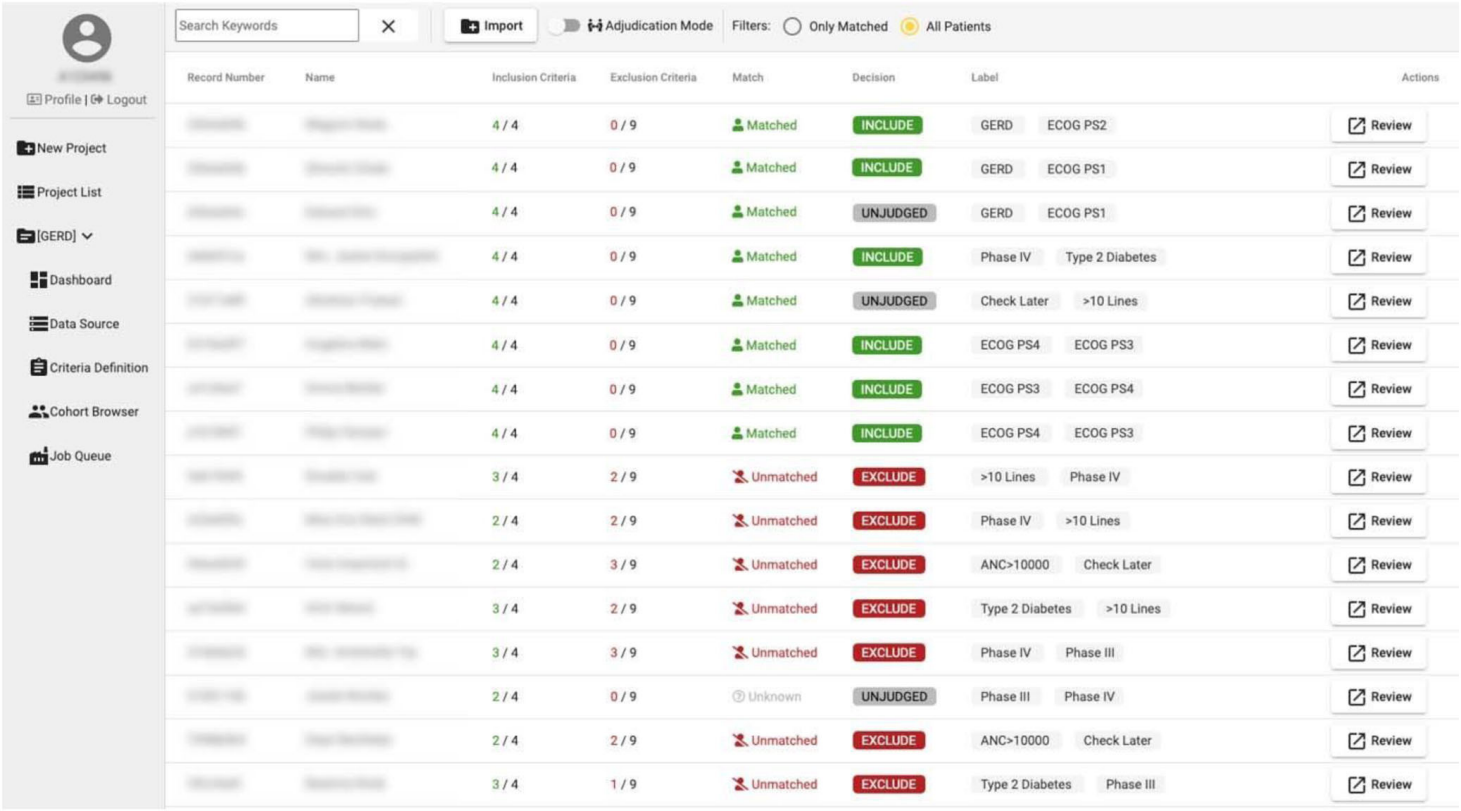
IMPACT patient accrual results page. A display of accrued patients that have been found to match a query phenotype definition (Fig. 3) in ranked order by closeness of match, alongside match status, abstraction/relevance judgement, and abstractor-supplied tags. An additional button to view matching criteria in more detail (Fig. 5) is also provided.

**Fig. 5:**
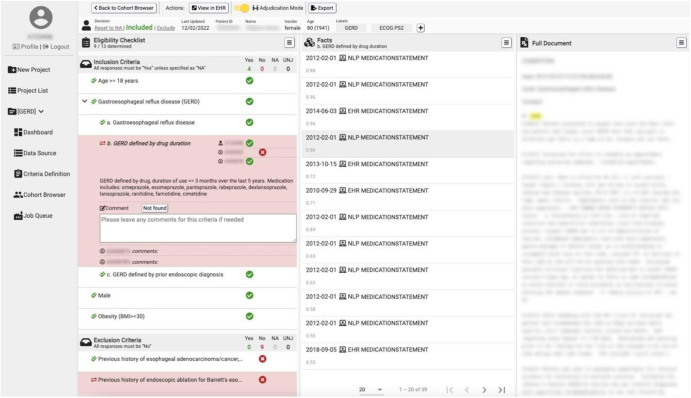
IMPACT evidence display page. A display of matching evidence by specific criteria elements. On the left pane, the query phenotype definition as a whole and whether a patient has been determined to match a given criterion is displayed. In the center, a listing of specific facts/evidence supporting a match/not match determination for the actively selected criterion is listed, with details on each individual fact/evidence item displayed on the right (including highlighted sections of clinical text, for NLP-based facts).

The web frontend offers an interface for phenotype definition (Fig. 3) and displays a list of patients sorted by match score (Fig. 4), with the option to switch to boolean filtering. Upon patient selection, the user is presented with the definition. The abstractor can view the evidence and judge their correctness for each definition criterion (Fig. 5). Switching to adjudication mode lists judgment conflicts between all abstractors.

These capabilities bring several benefits. Firstly, having the relevant evidence aggregated and presented to the adjudicator by matching phenotype criterion accelerates determination of whether a given patient matches the query phenotype. In addition, to perform iterative refinement and fine-tuning of phenotyping algorithms, algorithm errors (and evidence associated with said errors) must first be identified Having disagreement/adjudication functions built into the interface greatly facilitates this process. Finally, this interface/human-in-the-loop approach allows for the inclusion of external contextual information that may be absent from or contradict the clinical documentation itself, which may be helpful for certain use cases, e.g., “patient was contacted for a clinical trial, indicated that he had an undocumented positive/disqualifying smoking status”.

## Discussion

The desiderata presented here are not comprehensive: they are the results of our observations while implementing in-silico phenotyping, but experiences will vary. As such, we anticipate evolution in the framework as part of our open science efforts as feedback from users is incorporated. In addition, individual approaches to the various desiderata exist, but to our knowledge are spread across disparate toolsets and not integrated into a common solution. For example, while Atlas does offer phenotyping query execution, it is limited to using the OMOP CDM and does not support text retrieval. Similarly, EMERSE offers querying on text but has limited flexibility for working with multi-modal queries. Our current implementation is therefore intended to serve as a baseline that works reasonably and is easy to adopt/extend, but may not be state-of-the-art. To facilitate customization with other approaches, the application allows for modular component swapping.

A trade-off of infrastructure flexibility is runtime performance. Specifically, FHIR mapping is done on-demand to obviate instantiating a new data warehouse. Around 90%, per instrumentation, of runtime is spent on FHIR mapping. For reference, our observed performance using 128 central processing unit cores was 6 h for 1.9 million patients (with structured data and NLP). While this is still a significant improvement over manual efforts, pre-mapping/storing FHIR resources into a data store such as MongoDB or Elasticsearch, obviating on-demand mapping, would be more efficient.

Finally, while evaluations have previously been done on individual component implementations, a full evaluation in aggregate would be helpful. Due to the characteristics inherent to the phenotyping task, a meaningful systemic evaluation would require multiinstitutional deployment of the application and gold standard corpora development for each site across a variety of phenotyping tasks. For more details on this, please refer to the Supplementary Information. We have left such efforts to future work.

## Conclusions

Rapid in-silico clinical phenotyping on large datasets is of critical importance to accelerate research and development in the digital health domain. In this article, we have outlined some underlying complications hindering implementation of in-silico phenotyping and presented a framework, accompanied by an example implementation, addressing them.

## Supplementary information

Supplementary Information

## Data Availability

Data used as part of our use-case testing for the IMPACT implementation is considered protected health information and would be difficult to share with anyone not involved in an IRB-approved collaboration with the Mayo Clinic. We do, however, provide manually generated synthetic data that can be used as a stand-in to evaluate front-end GUI functionality. Said synthetic data is distributed alongside the IMPACT software application code.

## Code availability

The IMPACT implementation is open-source, code for which can be found at https://www.github.com/OHNLP/IMPACT. Please note that this repository is only the parent/tracking repository, and that IMPACT has several subcomponents each in their own GitHub repository. Links to the repositories for these subcomponents can be found in the README of the parent repository.

## References

[1] Weng C, Tu SW, Sim I, Richesson R (2010). Formal representation of eligibility criteria: a literature review. J. Biomed. Inf..

[2] Richesson RL, Horvath MM, Rusincovitch SA (2014). Clinical research informatics and electronic health record data. Yearb. Med. Inf..

[3] Thadani SR, Weng C, Bigger JT, Ennever JF, Wajngurt D (2009). Electronic screening improves efficiency in clinical trial recruitment. J. Am. Med. Inf. Assoc..

[4] Pathak J, Kho AN, Denny JC (2013). Electronic health records-driven phenotyping: challenges, recent advances, and perspectives. J. Am. Med. Inf. Assoc..

[5] Campion TR, Craven CK, Dorr DA, Knosp BM (2020). Understanding enterprise data warehouses to support clinical and translational research. J. Am. Med. Inf. Assoc..

[6] Ross J, Tu S, Carini S, Sim I (2010). Analysis of eligibility criteria complexity in clinical trials. Summit Transl. Bioinform..

[7] Madigan D (2013). Evaluating the impact of database heterogeneity on observational study results. Am. J. Epidemiol..

[8] Fu S (2022). Assessment of Data Quality Variability across Two EHR Systems through a Case Study of Post-Surgical Complications. AMIA Annu Symp. Proc..

[9] Elasticsearch B.V. *Elasticsearch*, https://github.com/elasticsearch/elasticsearch (2015).

[10] MongoDB Inc. *The MongoDB Database*, https://github.com/mongodb/mongo (2009).

[11] Google Inc. *BigQuery: Enterprise Data Warehouse*, https://cloud.google.com/bigquery (2011).

[12] Health Level 7 International. *Fast Healthcare Interoperability Resources (FHIR)*, https://hl7.org/fhir/R4/ (2019).

[13] Overhage JM, Ryan PB, Reich CG, Hartzema AG, Stang PE (2012). Validation of a common data model for active safety surveillance research. J. Am. Med. Inf. Assoc..

[14] Fleurence RL (2014). Launching PCORnet, a national patient-centered clinical research network. J. Am. Med. Inf. Assoc..

[15] Yadav, H., Du, Z. & Joachims, T. Policy-Gradient Training of Fair and Unbiased Ranking Functions. *Proceedings of the 44th International ACM SIGIR Conference on Research and Development in Information Retrieval.* ACM SIGIR 2021, 1044–1053 (2021).

[16] Hanauer, D. A. EMERSE: The Electronic Medical Record Search Engine. *AMIA Annu. Symp. Proc*. 2006 Annual Symposium of the American Medical Informatics Association, **941** (2006). PMC1839699 17238560

[17] Liu S (2020). Implementation of a Cohort Retrieval System for Clinical Data Repositories Using the Observational Medical Outcomes Partnership Common Data Model: Proof-of-Concept System Validation. JMIR Med. Inf..

[18] Apache Software Foundation. *Apache Lucene*, https://lucene.apache.org/ (2022).

[19] Shahi, D. *Apache Solr: A Practical Approach to Enterprise Search*. (APress, 2015).

[20] Wang Y (2018). Clinical information extraction applications: A literature review. J. Biomed. Inform..

[21] Fu S (2022). Ascertainment of Delirium Status Using Natural Language Processing From Electronic Health Records. J. Gerontol. A Biol. Sci. Med Sci..

[22] Sagheb E (2021). Use of Natural Language Processing Algorithms to Identify Common Data Elements in Operative Notes for Knee Arthroplasty. J. Arthroplast..

[23] Gao F (2018). SD-CNN: A shallow-deep CNN for improved breast cancer diagnosis. Comput Med. Imaging Graph..

[24] Sun, L. et al. Breast Mass Detection in Mammography Based on Image Template Matching and CNN. *Sensors (Basel)* **21** (2021). 10.3390/s21082855 10.3390/s21082855 PMC8072908 33919623

[25] Che H, Brown LG, Foran DJ, Nosher JL, Hacihaliloglu I (2021). Liver disease classification from ultrasound using multi-scale CNN. Int J. Comput. Assist Radio. Surg..

[26] Juhn YJ (2022). Assessing socioeconomic bias in machine learning algorithms in health care: a case study of the HOUSES index. J. Am. Med. Inf. Assoc..

[27] Obermeyer Z, Powers B, Vogeli C, Mullainathan S (2019). Dissecting racial bias in an algorithm used to manage the health of populations. Science.

[28] Rajkomar A, Hardt M, Howell MD, Corrado G, Chin MH (2018). Ensuring Fairness in Machine Learning to Advance Health Equity. Ann. Intern. Med..

[29] Moon S (2019). Salience of Medical Concepts of Inside Clinical Texts and Outside Medical Records for Referred Cardiovascular Patients. J. Health. Inf. Res..

[30] Kang T (2017). EliIE: An open-source information extraction system for clinical trial eligibility criteria. J. Am. Med. Inf. Assoc..

[31] Gilbert EH, Lowenstein SR, Koziol-McLain J, Barta DC, Steiner J (1996). Chart reviews in emergency medicine research: Where are the methods?. Ann. Emerg. Med..

[32] Fu S (2020). Assessment of the impact of EHR heterogeneity for clinical research through a case study of silent brain infarction. BMC Med Inf. Decis. Mak..

[33] Pagali, S. R., Kumar, R., Fu, S., Sohn, S. & Yousufuddin, M. Natural Language Processing CAM Algorithm Improves Delirium Detection Compared With Conventional Methods. *Am. J. Med. Qual.* (2022). 10.1097/JMQ.0000000000000090 10.1097/JMQ.0000000000000090 36283056

[34] Safarova MS, Liu H, Kullo IJ (2016). Rapid identification of familial hypercholesterolemia from electronic health records: The SEARCH study. J. Clin. Lipido..

[35] Zeng Z, Deng Y, Li X, Naumann T, Luo Y (2019). Natural Language Processing for EHR-Based Computational Phenotyping. IEEE/ACM Trans. Comput. Biol. Bioinform..

[36] Sohn S (2018). Clinical documentation variations and NLP system portability: a case study in asthma birth cohorts across institutions. J. Am. Med. Inf. Assoc..

[37] Bodenreider O (2013). The NLM value set authority center. Stud. Health Technol. Inf..

[38] Yuan C (2019). Criteria2Query: a natural language interface to clinical databases for cohort definition. J. Am. Med. Inf. Assoc..

[39] Liu H (2013). An information extraction framework for cohort identification using electronic health records. AMIA Jt Summits Transl. Sci. Proc..

[40] Savova GK (2010). Mayo clinical Text Analysis and Knowledge Extraction System (cTAKES): architecture, component evaluation and applications. J. Am. Med. Inf. Assoc..

[41] Vassar M, Holzmann M (2013). The retrospective chart review: important methodological considerations. J. Educ. Eval. Health Prof..

[42] Grishman R, Huttunen S, Yangarber R (2002). Information extraction for enhanced access to disease outbreak reports. J. Biomed. Inf..

[43] South BR (2009). Developing a manually annotated clinical document corpus to identify phenotypic information for inflammatory bowel disease. BMC Bioinforma..

[44] Anderson WP (2015). Reproducibility: Stamp out shabby research conduct. Nature.

[45] Baker D, Lidster K, Sottomayor A, Amor S (2012). Reproducibility: Research-reporting standards fall short. Nature.

[46] Begley CG, Buchan AM, Dirnagl U (2015). Robust research: Institutions must do their part for reproducibility. Nature.

[47] Kolker E (2013). Reproducibility: In praise of open research measures. Nature.

[48] Chapman WW (2011). Overcoming barriers to NLP for clinical text: the role of shared tasks and the need for additional creative solutions. J. Am. Med. Inf. Assoc..

[49] Musen MA, Rohn JA, Fagan LM, Shortliffe EH (1987). Knowledge engineering for a clinical trial advice system: uncovering errors in protocol specification. Bull. Cancer.

[50] Leung LY (2021). Agreement between neuroimages and reports for natural language processingbased detection of silent brain infarcts and white matter disease. BMC Neurol..

[51] Fu S (2020). Clinical concept extraction: A methodology review. J. Biomed. Inf..

[52] Observational Health Data Sciences and Informatics. *OHDSI/Atlas - an Open Source Software Tool for Researchers to Conduct Scientific Analyses on Standardized Observational Data*, https://github.com/OHDSI/Atlas (2022).

[53] Wu, S. et al. in *Proceedings of the 10th International Conference on Language Resources and Evaluation, LREC* 2016 3412-3416 (European Language Resources Association (ELRA), Portoroz, Slovenia, 2016).

[54] Apache Software Foundation. *Apache Beam*, https://beam.apache.org/ (2022).

[55] Zaragoza H, Robertson S (2009). The Probabilistic Relevance Framework: BM25 and Beyond. Found. Trends® Inf. Retr..

[56] Lv, Y. & Zhai, C. Lower-bounding term frequency normalization. *Proceedings of the 20th ACM international conference on Information and knowledge management*. CIKM '11, 7–16 (2011).

[57] Wen A (2019). Desiderata for delivering NLP to accelerate healthcare AI advancement and a Mayo Clinic NLP-as-a-service implementation. NPJ Digit. Med..

[58] Hong N (2018). Integrating Structured and Unstructured EHR Data Using an FHIR-based Type System: A Case Study with Medication Data. AMIA Jt Summits Transl. Sci. Proc..

[59] Hong N (2019). Developing a scalable FHIR-based clinical data normalization pipeline for standardizing and integrating unstructured and structured electronic health record data. JAMIA Open.

[60] Bodenreider O (2004). The Unified Medical Language System (UMLS): integrating biomedical terminology. Nucleic Acids Res..

[61] Observational Health Data Sciences and Informatics. *Athena: Observational Health Data Sciences and Informatics – OHDSI*, https://athena.ohdsi.org/ (2022).

