# Supplementary material for: The IMPACT framework and implementation for accessible in silico clinical phenotyping in the digital era
Source: NPJ Digit Med. 2023 Jul 21;6:132. doi: 10.1038/s41746-023-00878-9 (PMC10362064; doi:10.1038/s41746-023-00878-9)
Supplement: Supplementary file 1 — Supplementary Information [file 41746_2023_878_MOESM1_ESM.pdf]

With respect to horizontal scaling, Apache Beam, and associated frameworks, increased parallelization (i.e., running more than one computational task in parallel) is an oft-used strategy to reduce processing time for computational pipelines. This approach is particularly useful on large datasets when pipeline tasks can be processed relatively independently, minimizing the need for synchronizing computation between the different tasks, as is the case with in-silico patient phenotyping, where patient records can be scored independently for match against the phenotyping criteria and synchronization need only occur to rank the computed patient scores relative to one another.

Concurrently, there is a limit to the performance gained from parallelization on a single machine due to hardware bottlenecks. This is addressable via two differing scaling schemes: “vertical”, where the hardware itself to have greater capabilities, and “horizontal”, where additional machines are linked together (via networking) to tackle the problem. Past a certain point, however, vertical scaling becomes infeasible due to hardware simply not having the requisite capabilities. Conversely, horizontal scaling is limitless (so long as one has the financial ability to purchase more machines). The horizontal scaling paradigm has become especially popular given the advent of cloud computing: instead of paying for the costs to constantly maintain a computing cluster, additional computing capacity can be purchased on the fly and added to a computing cluster, and decommissioned once the capacity is no longer needed. Many scaling implementations also adopt a hybridized approach, where individual machines that are reasonably powerful are linked together in a cluster.

A substantial portion of practical tasks that one would wish to horizontally scale cannot, however, have their individual tasks made fully independent of one another. For instance, with the in-silico phenotyping step, while we can make scoring patients independent, the results must be eventually aggregated and compared to one another for ranking. Similarly, the source datasets themselves must be split amongst the different execution nodes and tasks assigned to each. This has led to a variety of cluster computing frameworks, ranging from Hadoop MapReduce, to its derivatives such as Apache Spark or Apache Flink, to cloud equivalents such as Google Cloud Platform’s Dataflow.

As a key desideratum is the need for infrastructure flexibility, and individual institutions have all adopted differing frameworks for their cluster computing environments, we elected to implement our backend using Apache Beam, which can directly translate execution into multiple scaling frameworks at runtime, including Apache Spark, Apache Flink and Google Cloud Platform’s Dataflow. Programs written with the Apache Beam framework can also be run locally on a single machine, for those wishing to do local debugging as well as for those using vertical scaling.

With respect to information retrieval relevance scoring and the BM25+ algorithm, information retrieval relevance scoring for text has traditionally been done using term frequency – inverse document frequency (TF-IDF) and derivative algorithms. This TF-IDF dual weight is useful as while a high document frequency for a certain query term does typically suggest that a document is more likely to be relevant to the user’s query, the prevalence of certain query terms within the overall collection differs. For instance, given a clinical phenotyping query for “some rare disease + hypertension”, it would be expected that the latter term would occur frequently in the overall document corpus, to include multiple instances (multiple encounters) for any given patient. In a purely TF-based ranking scheme the high individual term frequency per document (patient) could lead to patients with only hypertension (mentioned throughout several encounters) having a higher ranking than patients with both the rare disease in question and hypertension, as the rare disease would not be mentioned as often even within a single patient (particularly for patients with long clinical histories with the rare disease being diagnosed relatively recently). To counterbalance this, the IDF weight is used, where the score for terms that occur frequently throughout a document cohort is penalized relative to that of more rare terms.

Beyond TF-IDF, several derivatives following the same TF/IDF weighting scheme have been created designed to address various shortcomings in the base algorithm. Especially pertinent to the clinical

phenotyping use case is the Okapi BM25[55] variant, which further accounts for document length and term saturation. This is useful for the clinical phenotyping as the "document length" portion can be cast to "encounter/medical history length", where something occurring multiple times throughout a patient's medical history does not necessarily make a given patient more relevant to an end user. We therefore chose a BM25 variant, BM25+, which adds a lower bound on the term frequency by document length normalization, for our example implementation.

Default values as provided by Apache Lucene-for hyperparameters k_1, b, and δ are 1.2, 0.75, and 1.0 respectively. While these values have been shown to be reasonable defaults that offer suitable performance on many data collections, we have left hyperparameter values configurable by the end user should fine-tuning be desired. If fine-tuning is done, it is recommended that the hyperparameter tuning be done on a per-data source basis. This process is then repeated across all data sources, and scores integrated together via weighting followed by summation. By default, each data source is weighted equally, but depending on use case and institution level data documentation processes, the weights can be adjusted on a per-project basis via configuration.

With respect to the HL7 Fast Health Interoperability Resources (FHIR) [12], FHIR is a data standard intended to facilitate intersystem healthcare data exchange by ensuring that healthcare data is represented in a standardized format. This is particularly important in the case of cross-EHR data transfer as it ensures that the receiving system can correctly read/incorporate the clinical data sent by the originating EHR. Given that clinical information must also be represented internally to our application and exposed as part of an application programming interface (API) for end-user customizability of data sources, it is beneficial to use an existing data standard as opposed to creating our own.

We chose FHIR as our data representation for several reasons. Firstly, FHIR is a relatively complete (for major clinical entities of interest) data standard ensuring that it can be adopted with minimal additional overhead and to reduce the potential for needing to introduce non-standard data elements due to information not being representable in the existing standard. Additionally, our use case is similar to FHIR's originally intended use case, making it an especially relevant data standard to use: the API use case is essentially analogous to the cross-EHR data transfer use case, with IMPACT serving as the receiving system (as opposed to a destination EHR). Finally, due to the 21st century cures act, there has been substantial uptake of the FHIR data standard within healthcare institutions in the United States, increasing the likelihood that a mapping for clinical data sources already exists and can thus be adapted to our API. For instance, both the Epic and Cerner EHRs implement a FHIR API that can be used directly as a data source for IMPACT with minimal additional technical overhead/mapping work.

With respect to coding and entity linkage, the medical coding task, where individual diagnoses, medications, procedures, lab tests, etc. are transformed into some standardized codified form within a controlled vocabulary, is a common task in the medical domain. In the context of the in-silico phenotyping task, a significant portion of clinical information is codified in such a structured form within electronic data warehouses. It is therefore typical for in-silico phenotyping queries, particularly those that are SQL-based, to query via these codified representations rather than the raw textual descriptors themselves. Unfortunately, coding systems vary between data sources. While the various revisions of the International Classification of Diseases (ICD) codesets are commonly used in medical billing for diagnoses, many EHR representations instead internally use SNOMED Clinical Terms (SNOMEDCT) for representations of diagnoses. Similarly, coding systems for medications and lab tests differ between institutions, and institution-local coding systems being used is a common occurrence. In addition, common data models such as the OHDSI OMOP CDM commonly specify their own concept ids to medical concepts.

The Unified Medical Language System (UMLS) Metathesaurus provides a mapping between many of these differing vocabularies and is therefore a useful intermediary concept representation for toolsets that will potentially be deployed across a variety of environments and the associated disparate coding practices. For this reason, it is common for clinical natural language processing (NLP) pipelines to output to UMLS concept codes (commonly referred to as concept unique identifiers, or CUIs) for the named entity linking task instead of selecting a specific controlled vocabulary, particularly when potential outputs span multiple clinical semantic types, as is the case with our textual criterion parser. Output UMLS CUIs can then be mapped to whatever coding system is used locally in the data source using the UMLS-provided mappings.

With respect to system performance, system performance should be expected to vary significantly depending on a variety of factors including the complexity of the phenotype definition, the completeness and consistency of requisite data within the queried data sources, as well as the origin of the data used and associated impact on its contents. Additionally, such a performance number would typically be including human refinement, the extent of which will vary by task. Finally, the performance of individual components of the overall framework will vary based on many of these same factors between differing phenotyping tasks, further increasing performance variation.

Rather than presenting an overall system performance, we therefore instead present here general performance for our individual components, specifically for the three that are relevant to the retrieval task: textual criterion to data source representation mapping, BM25+ retrieval, and incorporation of multiple data sources in the scoring process.

In the former case, the NLP system used as part of IMPACT's example implementation has previously been evaluated to have a F1-score performance of 0.715 on clinical entities from clinical narratives and linking to appropriate UMLS CUIs[40].

In the latter two cases, information retrieval tasks have been primarily evaluated on pure text evaluation (e.g., for matching/searching biomedical articles by some phenotypic query, as is the case with TREC's 2018 precision medicine task, where BM25's observed performance was a Precision@10 of .56), but the incorporation of structured data and the associated change in data fidelity renders such a direct comparison unsuitable. Nevertheless, a preliminary study implementing much the same principles as the final IMPACT system incorporating both structured and unstructured data in scoring[17] suggests that inclusion of both types during scoring does help performance, with an average Precision@5 of 0.9 across 5 phenotyping queries as opposed to average of 0.5 and 0.7 for purely structured and purely unstructured data respectively.
